# Supplementary material for: Purification and Characterization of Authentic 30S Ribosomal Precursors Induced by Heat Shock
Source: Int J Mol Sci. 2023 Feb 9;24(4):3491. doi: 10.3390/ijms24043491 (PMC9959188; doi:10.3390/ijms24043491)
Supplement: Supplementary file 1 [file ijms-24-03491-s001.zip › ijms-2034875-supplementary.pdf]

Supplementary data

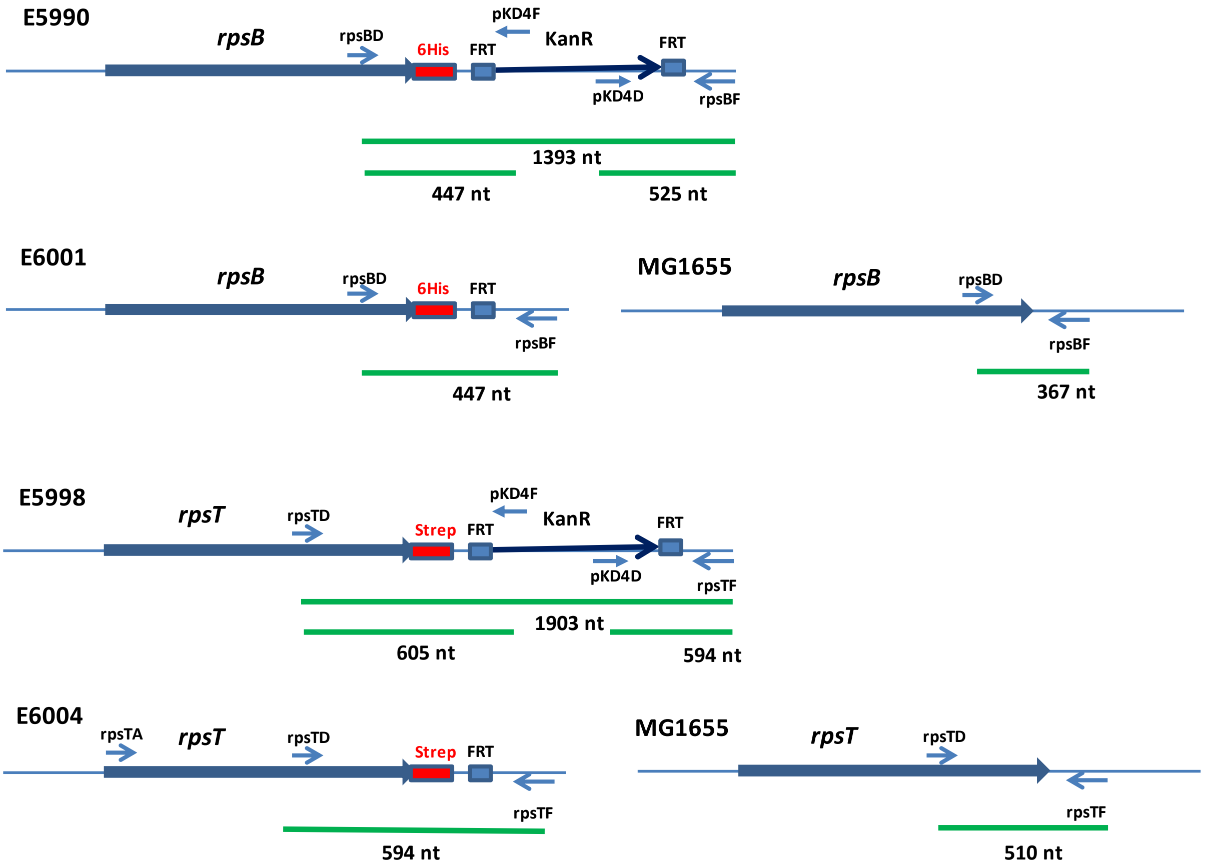


**Supplementary Figure S1. Verification of *rpsB*:his and *rpsT*:strep mutations.** The chromosomal DNA in strains E5990, E6001, E5998, E6004, and MG1655 was amplified by PCR using the indicated oligonucleotides (blue arrows). The size of the obtained PCR products is indicated below with green lines, and these results are in agreement with the predicted sizes.

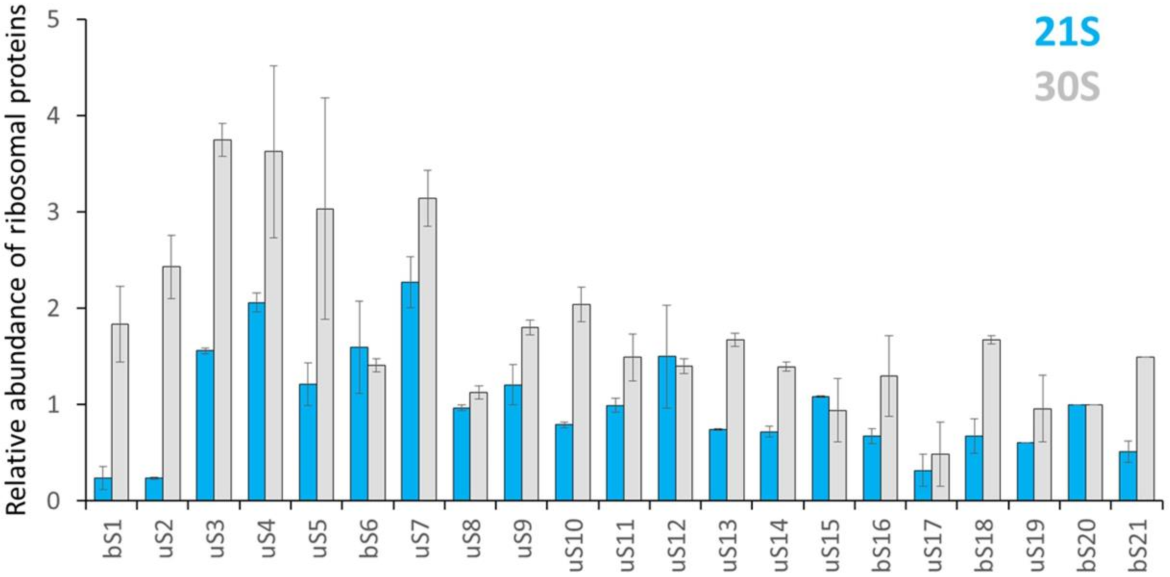


**Supplementary Figure S2. Relative quantification of 21S and 30S proteins by quantitative mass spectrometry.** Protein levels were normalized against the bS20 proteins used to purify the particles and are the means of three biological replicates, with standard deviation indicated.

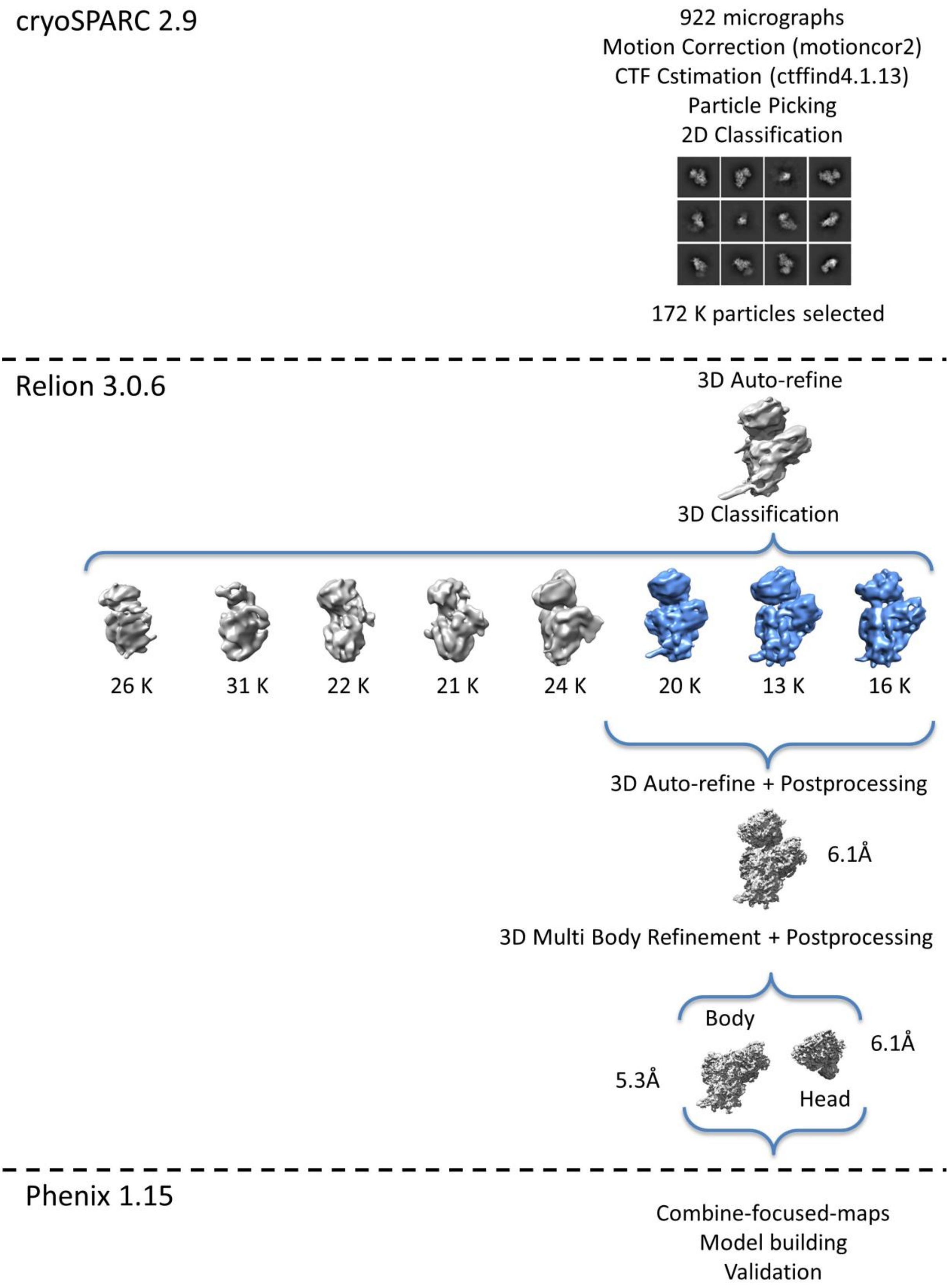


**Supplementary Figure S3. Summary of single-particle image analysis procedure**

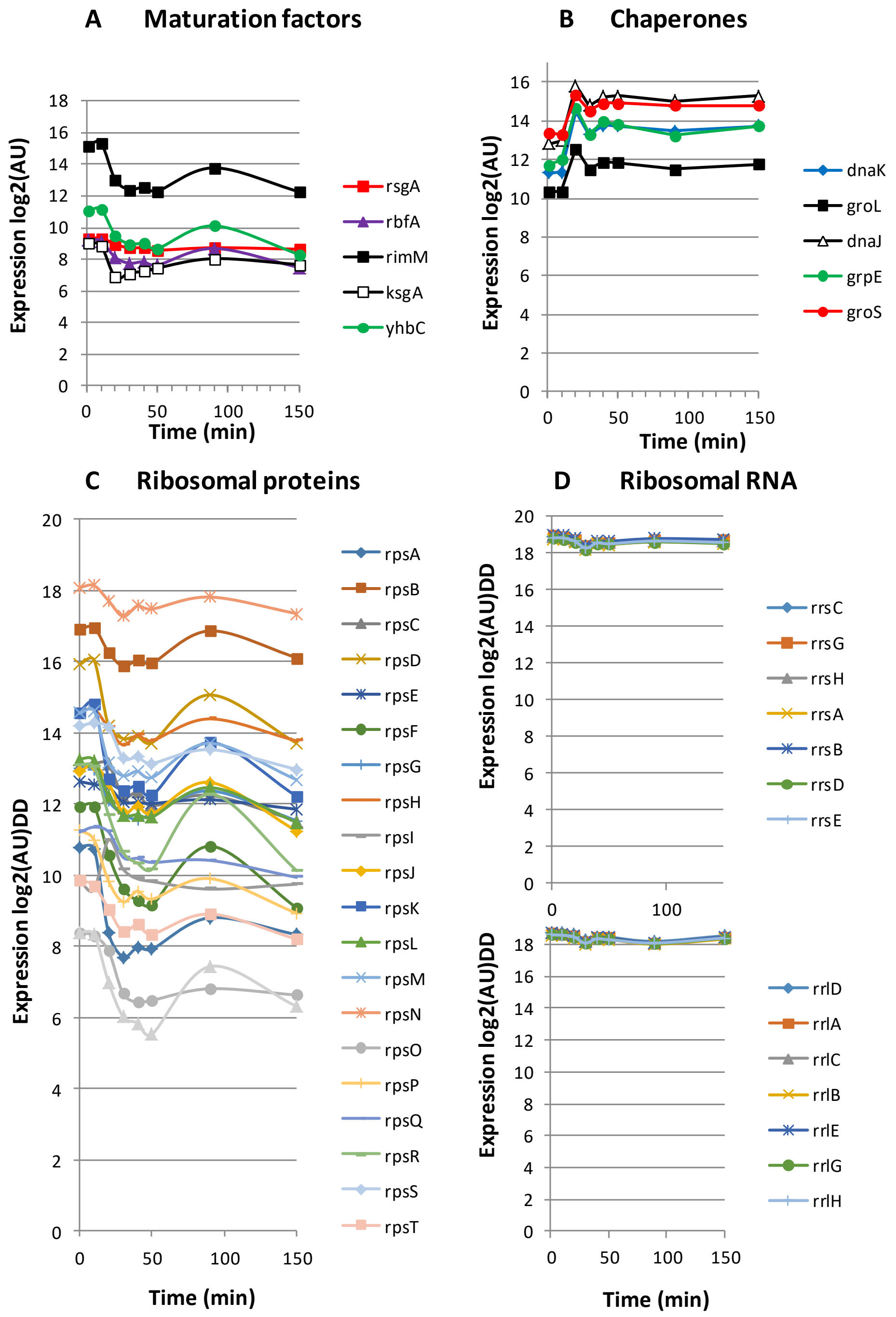


**Supplementary Figure S4. Varied gene expression after heat shock.** Data were extracted from *E. coli* (accession GSE20305) metabolomic and transcriptomic stress responses (Jozefczuk, Klie et al. 2010). Growth media and heat shock conditions were similar to those used in our study. Cells were grown in minimal medium, with glucose as the carbon source. Cultures were transferred from 37 °C to a water bath at 50 °C. While stirring, the temperature was raised to 45 °C in less than 2 minutes. After constant stirring at 330 rpm, the cultures were transferred to a water bath kept at 45 °C. For both temperature treatments, continual monitoring was done to ensure constant temperatures. Results are the means of three experiments.
